# The role of RGC degeneration in the pathogenesis of glaucoma

**DOI:** 10.7150/ijbs.103222

**Published:** 2025-01-01

**Authors:** Zhibo Si, Yuhang Fan, Mingxuan Wang, Jing Zhao, Yunmei Zhang, Dongmei Liu, Yajuan Zheng

**Affiliations:** 1 Department of Ophthalmology, The Second Hospital of Jilin University, Jilin University, Changchun 130000, Jilin, China.; 2 Department of Geriatrics, The Second Hospital of Tianjin Medical University, Tianjin Medical University, Tianjin 300000, China.; 3 Department of Ophthalmology, Changchun Yishidun Eye Hospital, Changchun 130000, Jilin, China.

**Keywords:** Glaucoma, Neurodegeneration, Retinal ganglion cells, neuroprotection, neurodegenerative diseases, Axon transport, Axon degeneration, Neuroinflammation

## Abstract

Glaucoma is a neurodegenerative disorder marked by the loss of retinal ganglion cells (RGCs) and axonal degeneration, resulting in irreversible vision impairment. While intraocular pressure (IOP) is presently acknowledged as the sole modifiable risk factor, the sensitivity of RGCs to IOP varies among individuals. Consequently, progressive vision loss may ensue even when IOP is effectively managed. This review consolidates current knowledge regarding the pathogenesis of RGCs in glaucoma and various neurodegenerative diseases. It delves into the mechanisms underlying RGC transsynaptic degeneration and axonal defects in glaucoma from a pathophysiological standpoint, and it elucidates the alterations in the visual pathway throughout the progression of the disease. Furthermore, the article outlines neuroprotective and nerve regeneration strategies aimed at vision restoration in glaucoma patients, offers insights for clinical management of the condition, and investigates prospective avenues for gene therapy.

## Introduction

Glaucoma is the leading cause of irreversible blindness globally. Data indicates that the global prevalence of glaucoma has risen to 76 million individuals in 2020, with projections suggesting a further increase to 120 million by 2040 1. The fundamental characteristic of this disease is progressive neurodegeneration, manifesting as axonal degeneration and the gradual loss of RGCs, which is accompanied by distinctive damage to the optic nerve and corresponding visual field deficits. Beyond the primary treatment of intraocular pressure reduction, additional factors influence the progression of RGC lesions. For instance, certain individuals with ocular hypertension do not experience significant optic nerve damage. For instance, certain individuals with ocular hypertension do not exhibit significant optic nerve damage. Conversely, some patients with glaucoma continue to experience progressive visual field loss despite maintaining intraocular pressure within the normal range. These observations indicate that there is variability in individual tolerance to intraocular pressure and susceptibility to glaucoma.

Hence, increasing evidence suggests that glaucoma extends beyond ocular manifestations to impact the visual pathway, encompassing the retina, lateral geniculate nucleus (LGN), and visual cortex, resembling degenerative disorders of the nervous system. Previous studies have identified glaucomatous damage in the lateral geniculate nucleus and visual cortex of glaucoma model monkeys. Furthermore, N Gupta's research team observed atrophy of the LGN and a decrease in cortical band thickness in comparison to the control group during their clinical pathological investigations of individuals with glaucoma 2. The debate on whether glaucoma initiates from the optic nerve and progresses upward, or originates from the central nervous system and subsequently affects the optic nerve and visual field, remains a prominent topic in contemporary research. Although retinal ganglion cell (RGC) apoptosis is recognized as the earliest form of cell death in glaucoma, studies have indicated that at least 25-35% of RGCs may be lost before damage is detectable through automated visual field testing. This finding supports the hypothesis of a descending onset of glaucoma, providing new evidence for this perspective. In recent years, research has increasingly focused on preventing vision loss in glaucoma patients by protecting the function of RGCs, based on studies of RGC dysfunction and death.

This review provides a comprehensive summary of central nervous system lesions observed in patients with glaucoma, with a particular focus on the role of RGCs in the disease's pathogenesis. Given the irreversible nature of RGC loss, this article systematically examines the interrelationship between glaucoma and the brain, investigates the shared pathophysiological mechanisms between glaucoma and central neurodegenerative diseases, and explores potential therapeutic directions for glaucoma from a neurodegenerative perspective, aiming to identify novel treatments that promote neuroprotection and neuroregeneration.

## Alterations in the visual pathways in individuals with glaucoma

The visual pathway comprises numerous neuronal chains tasked with conveying visual stimuli from the retina to the primary visual cortex. In humans, this pathway typically encompasses two distinct segments: the anterior visual pathway and the posterior visual pathway. As a component of the central nervous system, the human visual pathway is characterized by a range of 770,000-1,700,000 nerve fibers, corresponding to the number of retinal ganglion cell axons innervating each retina. RGCs, as neurons located in the retinal ganglion cell layer, play a crucial role in receiving visual stimuli from photoreceptors. Their axons form the optic nerve, optic chiasm, and optic tract, ultimately establishing synapses with the lateral geniculate body to facilitate the secondary transmission of visual information. This information is then relayed to the primary visual cortex in the cerebral cortex, where vision is processed.

**Figure 1:**
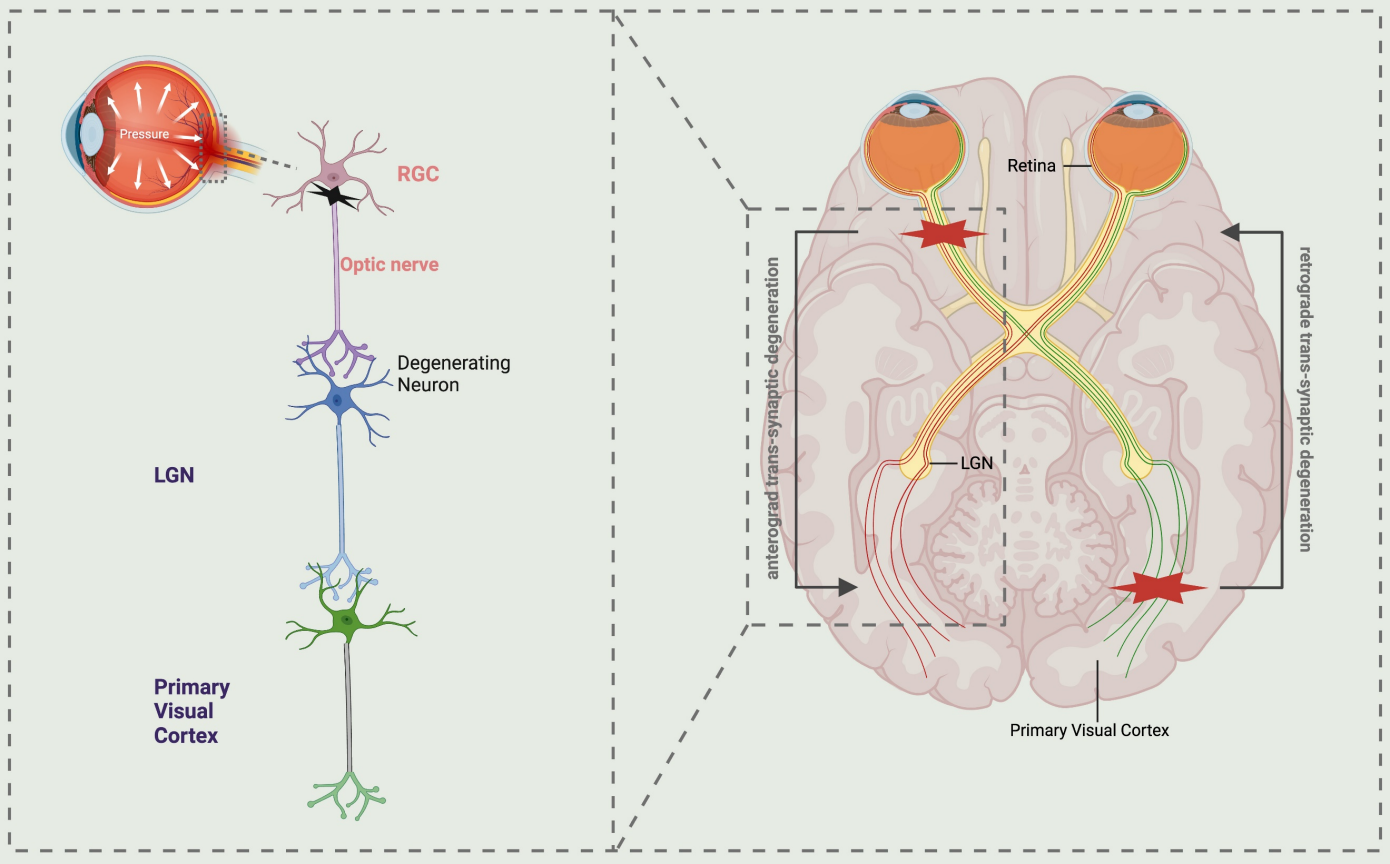
** Schematic representation illustrating the trans-synaptic degeneration of visual pathways in glaucoma.** The normal visual pathway is a well-functioning sensory system consisting of three levels of neurons, extending from the retina to the primary visual cortex. Atrophy of retinal ganglion cells (RGCs) in glaucoma leads to anterograde trans-synaptic degeneration along the optic nerve, lateral geniculate nucleus (LGN), and the optic radiations extending to the primary visual cortex. While retrograde trans-synaptic degeneration refers to the process of retinal degeneration that occurs as a consequence of damage to the posterior visual pathway. LGN lateral geniculate nucleus, RGCs retinal ganglion cells.

Numerous contemporary research studies on neurodegeneration in glaucoma have demonstrated that axonal injury propagates along the optic nerve to the posterior visual pathway, resulting in degeneration of the visual cortex, referred to as anterograde degeneration. Conversely, the progression from postsynaptic to presynaptic neurons is termed retrograde degeneration. Additionally, trans-synaptic degeneration (TSD) is a phenomenon characterized by sexual degeneration 3, 4. Therefore, TSD is regarded as the underlying mechanism for overall tissue degeneration in a range of neurodegenerative disorders (Fig. 1). Initially, research conducted on primate experimental animals revealed that chronic elevation of intraocular pressure is correlated with a reduction in the volume, density, and quantity of neurons within the lateral geniculate nucleus 5. Subsequently, a notable degeneration of the magnocellular and parvocellular layers within the lateral geniculate body, which transmits information to the visual cortex, was observed in a glaucoma monkey model 6, 7. Since the initial literature report in 2006 detailing the reduction of brain cortical volume in individuals with advanced glaucoma, subsequent studies have confirmed the impact of glaucoma on brain synapses within the visual pathway 2. Murphy utilized functional magnetic resonance imaging (MRI) to observe diminished cortical activity in glaucoma patients relative to healthy controls. This finding, in conjunction with standard automated visual field examination, suggests that despite reduced cortical activity, visual field function remains within normal parameters. However, beyond a certain threshold, a decline in visual function can be detected 8. This finding has the potential to offer valuable assistance and direction for the prompt identification and diagnosis of glaucoma. Moreover, the utilization of proton magnetic resonance spectroscopy in both animal models and glaucoma patients has yielded consistent evidence suggesting a potential association between cholinergic and glutamatergic abnormalities and the degeneration of the visual cortex in glaucoma 8-11. Currently, the cholinergic system and citicoline have shown promise as neuroprotective agents for the treatment of neurodegenerative conditions such as Alzheimer's disease and Parkinson's disease 12-14. Based on this hypothesis, targeting citicoline for assessment and treatment of glaucoma neurodegeneration may potentially enhance the visual acuity of individuals with glaucoma.

## Pathophysiology of glaucoma

### Mechanical theory

**Figure 2:**
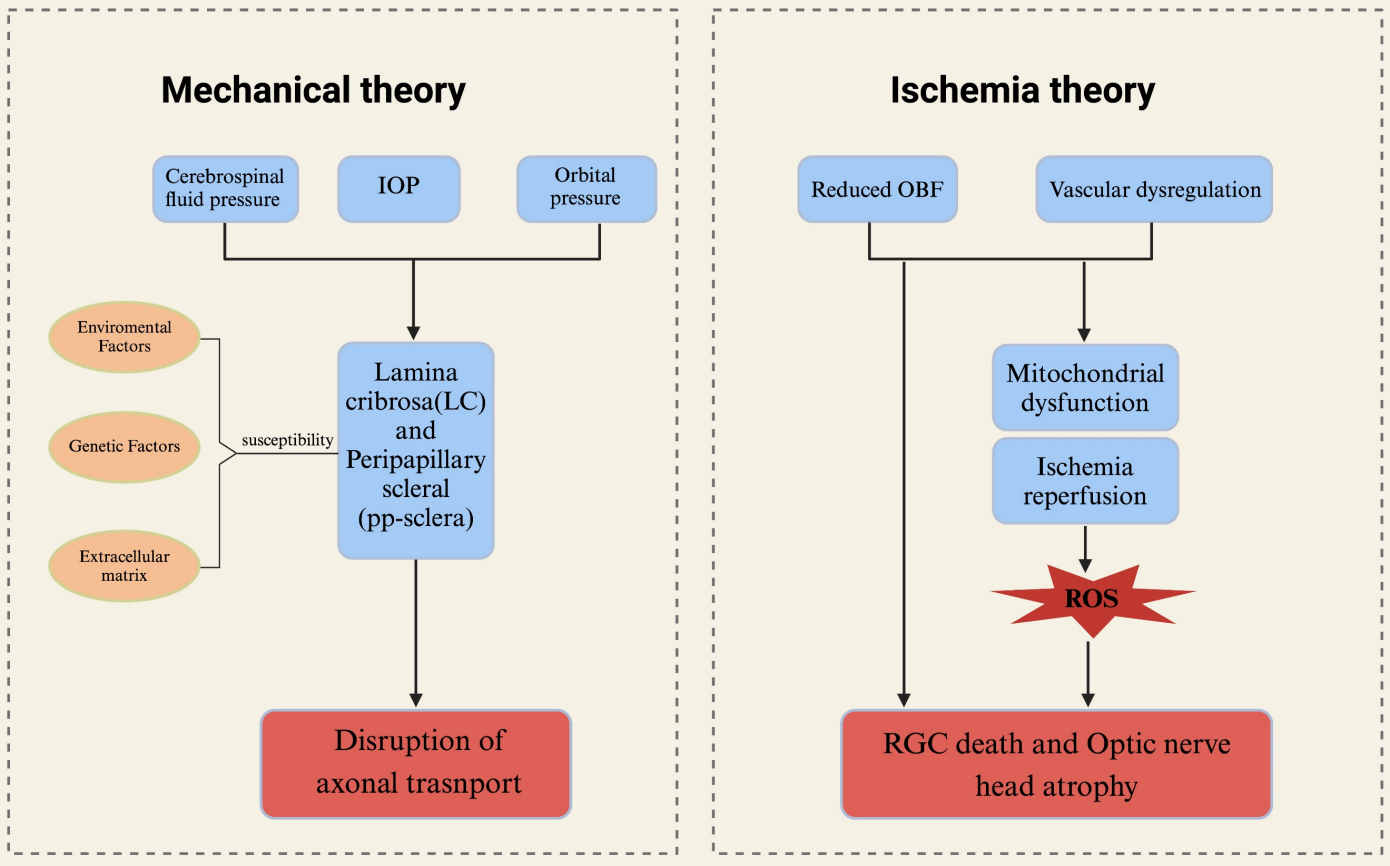
** The mechanical theory and ischemic theory in the physiology of glaucoma.** The primary connective tissue components constituting the optic nerve head (ONH) are the lamina cribrosa and the surrounding sclera. These structures may be influenced by intraocular pressure, cerebrospinal fluid pressure, and orbital pressure. The characteristics of connective tissue exhibit significant interindividual variability, influenced by environmental factors, genetic predispositions, and the composition of the extracellular matrix, all of which contribute to the susceptibility of axons. These mechanical factors can potentially result in the loss of retinal ganglion cells (RGCs) and the disruption of axonal transport. The ischemic theory of glaucoma posits that primary damage occurs in the ONH. Insufficient ocular blood flow and/or disordered vascular autoregulation directly result in the death of RGCs and atrophy of the ONH. Concurrently, ONH ischemia due to reduced blood flow can lead to mitochondrial dysfunction. Alterations in ocular blood flow (OBF) and elevated intraocular pressure impair vascular autoregulation, inducing ischemia-reperfusion injury. This triggers the generation of reactive oxygen species (ROS) and oxidative stress, further exacerbating damage to RGCs. ONH optic nerve head, OBF ocular blood flow, ROS reactive oxygen species.

The convergence of axons from RGCs to form the optic nerve occurs in the optic disc region. In primates, these axons are demyelinated as they pass through the lamina cribrosa. The mechanistic theory of glaucoma pathophysiology posits that sustained elevation of intraocular pressure leads to compression of the optic nerve, structural changes in the lamina cribrosa, alterations in synaptic transmission of neurons, and disruption of retrograde transport of neurotrophic factors. And the lamina cribrosa is recognized as the principal location of optic nerve damage in glaucoma, rendering it a vulnerable site for RGCs and associated with alterations in the visual field 15, 16. Although elevated intraocular pressure is widely recognized as the primary risk factor for glaucoma, individual variances exist, leading to varying levels of susceptibility to intraocular pressure-induced damage. This phenomenon is exemplified in cases of normal tension glaucoma, where structural disparities in the optic nerve head and lamina cribrosa may play a significant role 17. The aforementioned concept was substantiated through a clinical investigation involving glaucoma patients over a follow-up duration of up to 5 years. Utilizing spectral domain optical coherence tomography (SD OCT), the optic nerve head (ONH) and lamina cribrosa were assessed in these patients. The study revealed a notable displacement of the ONH and anterior lamina cribrosa (the opening of Bruch's membrane) in a substantial proportion of patients, with the extent of displacement being correlated with intraocular pressure levels during the follow-up period 18. When subjected to prolonged mechanical stress, the optic nerve head (ONH) will experience alterations in both morphology and microstructure, which are thought to impact both anterograde and retrograde axonal transport. The preservation of proper axonal transport is essential for the maintenance of axonal function, with RGCs potentially being more vulnerable to transport disruptions due to their elongated axonal morphology 19, 20 (Fig. 2). Of course, there are numerous potential factors contributing to axonal transport impairment in glaucoma, such as ischemia, neuroinflammation, and oxidative stress 21-23.

### Ischemia theory

The current research on the ischemic theory of glaucoma primarily focuses on the insufficient blood supply to the ophthalmic artery, which is hypothesized to be a potential pathogenic mechanism independent of elevated intraocular pressure. This includes conditions such as open-angle glaucoma, where the autoregulation of retinal blood vessels is impaired and inadequate. Additionally, blood reperfusion is believed to have a detrimental impact on glaucomatous optic neuropathy. Currently, various technologies have demonstrated a correlation between glaucoma and diminished ocular blood flow. Research indicates that reduced ocular blood flow in the optic nerve head (ONH) may contribute to the progression of glaucoma. The development of non-invasive measurement techniques for ocular blood flow (OBF), including optical coherence tomography angiography (OCTA), color Doppler imaging, and laser speckle flowgraphy (LSFG), enables precise assessment of blood flow in the ONH, retina, and choroid 24. In a study employing Laser Speckle Flowgraphy (LSFG) to evaluate optic nerve head (ONH) blood flow in nonhuman primates, a significant reduction in blood flow was observed in eyes with early-stage glaucoma. Additionally, vasoconstriction in both the large vessel area and the capillary area of the ONH was detected during the preclinical stage of glaucoma 25, 26. These findings collectively suggest that diminished ocular blood flow (OBF) may be a primary factor contributing to the pathophysiological changes observed in the early stages of glaucoma.

Vascular factors are deemed highly significant in the context of normal-tension glaucoma (NTG). Numerous studies have identified hypotension, primary vascular disorders, and circadian fluctuations in mean ocular perfusion pressure as principal risk factors for NTG. Additionally, research has substantiated that the prevalence of obstructive sleep apnea syndrome (OSAS) is markedly higher among individuals with NTG 27, 28.

The optic nerve head exhibits a high sensitivity to alterations in the blood supply from the ophthalmic artery. Consequently, ischemia-reperfusion injury and chronic oxidative stress, resulting from inadequate blood supply, induce modifications in the microenvironment, ultimately leading to the loss of RGCs. Research has demonstrated a significant accumulation of mitochondria at the lamina cribrosa, the region where RGC axons exit the eye and undergo demyelination 29. Mitochondrial dysfunction holds substantial importance in the context of glaucomatous optic neuropathy, primarily due to the elevated energy requirements associated with axonal transport and transmission in this region. This dysfunction elucidates the heightened susceptibility observed with advancing age, which is attributed to a reduction in mitochondrial quantity and an increase in mitochondrial DNA (mtDNA) mutations, as identified in primary open-angle glaucoma (POAG) 30, 31.

## Glaucoma and neurodegenerative diseases

### Glaucoma and Alzheimer's disease

Alzheimer's disease (AD) represents the most prevalent form of dementia among the elderly, primarily distinguished by the extracellular accumulation of amyloid-β (Aβ) and the intracellular deposition of hyperphosphorylated tau. Both AD and glaucoma are neurodegenerative disorders that are characterized by synaptic dysfunction and the excessive accumulation of aberrant proteins.

Alzheimer's Disease (AD) patients frequently exhibit ocular manifestations, including impaired contrast sensitivity, visual integration disorders, and visual field defects. Studies have indicated that individuals with AD may experience a reduction in RGCs, particularly in the fovea, as well as optic nerve axon degeneration and elevated amyloid-beta levels in the aqueous humor. These visual impairments are hypothesized to result either directly from cortical degeneration or indirectly from retinal inflammation and degeneration induced by chronic oxidative stress associated with iron homeostasis disorders. In post-mortem examinations of the retinas of Alzheimer's disease (AD) patients, it was observed that, in addition to the loss of RGCs, there was a concomitant reduction in melanopsin retinal ganglion cells (mRGCs), which are known to play a crucial role in the regulation of circadian rhythms 32. Furthermore, amyloid-beta (Aβ) deposition in the retina, extending from the outer nuclear layer (ONL) to the nerve fiber layer (NFL), was found to occur concurrently with cerebral amyloid pathology 33-35.

Glaucoma and Alzheimer's disease are hypothesized to share certain commonalities, notably the irreversible degeneration of nerve cells. The amyloid precursor protein (APP) is coexpressed in both ocular and cerebral tissues and undergoes translation and processing via two distinct pathways: the non-amyloidogenic and amyloidogenic pathways. In the amyloidogenic pathway, APP is sequentially cleaved by β-secretase and γ-secretase, resulting in the production of amyloid-beta (Aβ) fragments of varying lengths. Among these fragments, Aβ42 is particularly neurotoxic and constitutes a major component of senile plaques 36. The accumulation of extracellular amyloid-beta (Aβ), observed in both the retina and brain, may contribute to disease progression through mechanisms of synaptic dysfunction 37. Concurrently, studies utilizing a rat model of glaucoma have demonstrated that retinal Aβ levels escalate in response to elevated intraocular pressure, increased light exposure, and aging 38. Furthermore, neuroinflammation triggered by aberrant proteins constitutes a critical aspect of the pathogenesis in both conditions. The accumulation of amyloid-beta (Aβ) deposits prompts the activation of microglia and astrocytes. These activated glial cells subsequently release a spectrum of anti-inflammatory and pro-inflammatory mediators. Following neuronal injury, this activation induces an inflammatory response in microglia, enhancing their phagocytic and migratory capabilities 39. Experimental animal models of glaucoma have demonstrated that defects in CX3CR1 activation lead to increased microglial activity, heightened neurotoxicity, and subsequent retinal ganglion cell (RGC) death 40. In addition, the overexpression of γ-synuclein has been observed in glial cells and optic nerve axons in patients with glaucoma, corroborating the role of neuroinflammation in the degenerative changes of neurons associated with this condition 41. Consequently, from the standpoint of synaptic transmission within the visual pathway, the degenerative damage to RGCs not only disrupts the input function from photoreceptors but also impairs the subsequent transmission of visual information to the brain.

### Glaucoma and Parkinson

Parkinson's disease is widely recognized as a progressive neurodegenerative disorder characterized by pronounced motor dysfunction and a variety of non-motor symptoms. These clinical manifestations are associated with the progressive degeneration of dopaminergic neurons in the substantia nigra, which has been linked to the pathological accumulation of intracellular alpha-synuclein. As the disease advances, there is a notable accumulation of aggregates composed of this protein, known as Lewy bodies. In particular, postmortem analyses of patients with Parkinson's disease have revealed the presence of α-synuclein deposits in neurons across various retinal layers, including the inner nuclear layer, inner plexiform layer, and ganglion cell layer 42, 43. Ocular manifestations, including hallucinations, disrupted circadian rhythms, and color vision deficiencies, are prevalent in approximately 80% of patients with Parkinson's disease. These symptoms are likely associated with retinal ganglion cell abnormalities detected through electroretinography 44. Evidence suggests that these ocular manifestations in Parkinson's disease patients are attributable to inflammation of the retinal nerve fiber layer (RNFL) cells and degeneration of the perifoveal dopaminergic plexus 45. In an 8-year longitudinal study involving patients diagnosed with open-angle glaucoma (POAG), Alzheimer's disease (AD), and Parkinson's disease (PD), it was observed that a diagnosis of POAG was associated with an increased risk of developing AD, but not PD 46. Concurrently, non-invasive measurement techniques, such as Optical Coherence Tomography (OCT) and Optical Coherence Tomography Angiography (OCTA), revealed that patients with PD exhibited alterations in retinal microvasculature and a reduction in the thickness of the retinal nerve fiber layer when compared to age- and gender-matched control subjects 47. From an etiological standpoint, the retina can be considered an extension of the brain, with neuroinflammatory responses posited to play a significant role in ocular neurodegenerative diseases. Regarding pathogenic mechanisms, both glaucoma and Parkinson's disease are characterized by the progressive degeneration of specific neuronal populations. This degeneration may arise from individual or synergistic effects, including oxidative stress, neuroinflammation, and mitochondrial dysfunction.

## Degeneration of neurons within the central visual system associated with glaucoma

Previous studies have demonstrated that glaucoma exhibits substantial pathophysiological parallels with degenerative disorders of the central nervous system. Notably, both anterograde and retrograde transsynaptic degeneration are observed in glaucoma, leading to the degeneration of RGCs and subsequent atrophy of the optic nerve, optic tract, lateral geniculate nucleus, primary visual cortex, and other associated structures within the visual pathway 48. In recent years, advancements in neuroimaging technology have facilitated an enhanced focus on the morphological and functional alterations within the visual pathway of individuals diagnosed with glaucoma 49.

### Lateral geniculate nucleus

The lateral geniculate nucleus (LGN), located within the thalamus, is integral to the visual pathway. Upon exiting the optic nerve, axons of human retinal ganglion cells converge at the optic chiasm to form the optic tract. Approximately 90% of these axons terminate in the LGN, whereas the remaining 10% project to other structures, including the superior colliculus and anterior tectum. The lateral geniculate nucleus (LGN) serves as a crucial intermediary in the visual processing pathway, receiving raw visual input from the retina and subsequently transmitting this information to the primary visual cortex, specifically the V1 region. The lateral geniculate nucleus (LGN) is present bilaterally within the cerebral hemispheres and is organized into six distinct cell layers. The magnocellular layer is situated in the ventral layers (1 and 2), while the parvocellular layer is situated in the dorsal layers 3-6. Each layer contains both M cells and P cells, which are interspersed by Koniocellular cells. LGN layers 2, 3, and 5 predominantly receive synaptic inputs from the ipsilateral eye, while layers 1, 4, and 6 primarily receive synaptic inputs from the contralateral eye 50, 51. Various cellular layers within the visual system execute specialized functions in the processing of visual information. Specifically, the magnocellular layer is implicated in the processing of visual information pertinent to motion detection and spatial resolution. In contrast, the parvocellular layer receives input from cone photoreceptors, exhibits sensitivity to high spatial frequencies, and predominantly mediates red-green color perception. The precise function of the K cell layer remains indeterminate, however, emerging evidence suggests it may be more attuned to blue-yellow color processing 52.

Recent evidence indicates that target neurons within the lateral geniculate nucleus (LGN) undergo degeneration, atrophy, and eventual cell death in both experimental glaucoma models and clinical cases. Notably, the parvocellular (P) cell layer primarily exhibits neuronal atrophy, whereas the magnocellular (M) cell layer predominantly experiences cell loss and atrophy affecting both the M and P pathways. The severity of this damage is directly correlated with the loss of RGCs, with atrophy and loss in the M cell layer occurring earlier than in the P cell layer 5. The k-cell layer, located between the m and p-cell layers, contains neurons that specifically express the calcium/calmodulin-dependent kinase type II-α (CaMKII-α isoform). In a study utilizing a glaucoma monkey model, a significant reduction in CaMKII-α immunoreactivity was observed in monkeys subjected to prolonged elevated intraocular pressure, in comparison to the control group. Notably, a substantial decrease was detected even in monkeys displaying minimal or no retinal ganglion cell (RGC) axon loss, and no direct correlation was found between the extent of CaMKII-α neuron loss and the degree of RGC axon loss. This phenomenon may be attributed to the association of CaMKII-α immunoreactivity with elevated intraocular pressure preceding RGC death 53, 54. Furthermore, numerous studies have reported increased apoptosis and autophagy within the lateral geniculate nucleus in glaucoma models. These findings are corroborated by the identification of apoptotic bodies and autolysosomes in specific neurons, as evidenced by transmission electron microscopy 55.

Previous research has identified the deposition of amyloid beta (Aβ) and tau proteins in the glaucomatous retina as a potential connection between glaucoma and neurodegenerative diseases. A recent study utilizing a monkey model of chronic glaucoma, induced via laser photocoagulation, has demonstrated the presence of Alzheimer's disease-like deposits of amyloid beta and phosphorylated tau (p-Tau) in the lateral geniculate nucleus 56. This study identified the presence of neuroinflammatory plaques composed of dystrophic neurons aggregated extracellularly, along with significant myelin sheath swelling in the glaucomatous lateral geniculate nucleus (LGN) compared to control samples. The intermediate conformation of amyloid beta, particularly Aβ1-42, is currently recognized as a cytotoxic agent. Furthermore, dysfunction of the phosphorylated tau (p-tau) protein impairs the intracellular transport of nutrients, leading to axonal transport defects 57, 58. The observation of Alzheimer's disease-like pathological changes in the lateral geniculate nucleus of individuals with glaucoma, consequent to retinal ganglion cell damage, further substantiates the robust association between glaucoma and Alzheimer's disease. Inhibiting the formation and accumulation of Alzheimer's disease-related proteins may offer a promising therapeutic strategy for glaucoma. This underscores the critical importance of identifying neuroprotective targets for effective treatment.

### Primary visual cortex

Neurons within the lateral geniculate nucleus are integral to the processing and analysis of visual stimuli, giving rise to extensive optic radiations that project to the primary visual cortex, also known as area V1. These nerve fibers, collectively termed the optic radiation bundle, form a crucial connection between the lateral geniculate nucleus and the primary visual cortex. The optic radiation is further subdivided into superior and inferior divisions, which respectively target the upper and lower regions of the visual cortex, thereby conveying information from the corresponding upper and lower visual fields 59, 60. The V1 area is organized into six distinct cellular layers, arranged sequentially from the outermost to the innermost layer. Among these, the fourth layer plays a pivotal role in information processing and is further divided into four sub-layers: 4Cα, 4Cβ, 4A, and 4B. Sub-layers 4Cα and 4Cβ predominantly receive input from the lateral geniculate nucleus, whereas sub-layers 4A and 4B primarily receive input from other cortical regions.

Previous studies have documented significant structural and functional anomalies in the primary visual pathways of individuals diagnosed with glaucoma. Recent advancements in detection technology now facilitate the visualization of these alterations and impairments in the visual pathways of glaucoma patients through sophisticated imaging techniques. A study employed blood oxygen level-dependent (BOLD) functional MRI to evaluate cortical responses to stimulation in participants with differing severities of glaucoma. The results demonstrate that increased disease severity is associated with reduced BOLD activity in the retinal mapping area of the visual cortex in response to visual field stimulation. Notably, the primary visual cortex exhibited more pronounced impairment relative to higher-order cortical regions 8. Utilizing functional MRI, the study observed a decrease in cerebral gray matter volume and cerebral blood perfusion in individuals with advanced primary open-angle glaucoma (POAG) compared to control subjects, aligning with prior findings suggesting potential cerebrovascular insufficiency in glaucoma 11, 61-63. Moreover, abnormalities in cerebral blood flow (CBF) are frequently observed in patients with Parkinson's disease and Alzheimer's disease, in addition to glaucoma, indicating that cerebrovascular dysfunction may play a contributory role in central neurodegenerative diseases 64-66. But the exact relationship between neurovascular alterations in glaucoma and brain degenerative processes remains unclear. It is plausible that disruptions in cerebrovascular responses lead to insufficient oxygen delivery to neurons, thereby facilitating the production of reactive oxygen species, impairing mitochondrial function, and causing redox imbalances 64.

Yang *et al.* conducted a study using mouse brain slices to quantify neuronal populations in glaucoma-afflicted DBA/2J mice. Utilizing NeuN and Nissl bodies as neuronal markers, they observed a diminished density of Nissl corpuscles in the V1 region of DBA/2J mice relative to control groups, suggesting a reduction in neuronal numbers within the visual cortex 67. In a separate rat model of glaucoma, Ailen *et al.* substantiated the impact of glaucoma on mitochondrial ATP production within the primary visual cortex. Specifically, impairment to complex II of the mitochondrial respiratory chain results in a reduction of ATP production and an elevation in reactive oxygen species generation 68. Inadequate mitochondrial antioxidant defenses can precipitate oxidative damage to lipids and proteins within mitochondria, culminating in a redox imbalance. The maintenance of mitochondrial function is critically important in the context of central nervous system disorders. The association between mitochondrial dysfunction-induced neuronal damage and loss, and various neurodegenerative diseases, has been extensively documented. Consequently, therapeutic strategies focused on modulating mitochondrial function have emerged as a significant area of investigation in contemporary scientific research 69-71.

## RGCs in Glaucoma

RGCs are specialized neurons responsible for conveying visual information from the retina to the brain. Similar to other neurons within the central nervous system, the demise of RGCs is irreversible. A multitude of risk factors and molecular mechanisms converge to precipitate the degeneration and subsequent death of RGCs.

### The crucial role of RGCs in glaucoma

In the retina, the transmission of information by RGCs is distinctive because the signal transduction from photoreceptors to bipolar neurons and subsequently to RGCs occurs without myelination. However, upon passing through the optic nerve head, the axons of RGCs become myelinated, and this myelination persists as the axons continue toward the central regions of the brain 72. It is consequently widely accepted that retinal ganglion cell (RGC) axons are particularly vulnerable to pathological stressors during the initial stages of disease progression. This susceptibility can be attributed to the axons' exceedingly high metabolic demands, necessitating substantial energy resources to sustain anterograde transport from the retina to the brain, retrograde transport from the brain to the retina, and transsynaptic transport between cells 73, 74. The formation of myelin enhances axonal efficiency by reducing energy consumption. Consequently, unmyelinated regions exhibit lower efficiency and necessitate greater energy expenditure to sustain conduction. This increased energy demand is evidenced by the higher density of mitochondria observed in the unmyelinated segments of RGCs 75, 76. The maintenance of the optic nerve head necessitates elevated energy levels. Approximately 70% of the mitochondria within the optic nerve are situated in astrocytes 75. Mitochondrial transport occurs in an anterograde or retrograde manner along tubulin and actin tracks in response to physiological signals^77^. Consequently, any condition that impacts mitochondrial function could result in the accumulation of free radicals, increased mitochondrial permeability, and other related effects 78-80.

### Physiological changes in RGC cell bodies

#### Glutamate toxicity in RGCs

Glutamic acid serves as the principal excitatory amino acid within the retina. Excitotoxicity arises from elevated levels of endogenous glutamate and the subsequent overactivation of glutamate receptors, resulting in an influx of calcium ions that initiates a cascade of biochemical reactions. This process ultimately culminates in the degeneration and apoptosis of RGCs 81. Historically, it was hypothesized that elevated glutamate levels were a requisite factor in the excitotoxic damage associated with glaucoma. However, contemporary research indicates that this is not the case. Throughout the chronic and progressive course of glaucoma, glutamate levels do not exhibit a significant increase or accumulation. Instead, glutamic acid may experience localized elevation in specific regions of the optic nerve, and extracellular glutamate levels may undergo small, persistent fluctuations. These subtle changes could contribute to the progression of glaucoma 82, 83. Under typical physiological conditions, Müller cells regulate extracellular glutamate concentrations by absorbing excess glutamate through the glutamate/aspartate transporter (GLAST or EAAT1) and subsequently converting it to glutamine via the glutamine synthetase. In glaucoma, the glutamate clearance system is compromised as a result of oxidative stress, damage to Müller cells, or dysfunction of the GLAST. This impairment leads to increased extracellular glutamate concentrations in the retina, particularly in proximity to RGCs 84. Elevated extracellular concentrations of glutamate lead to the overstimulation of NMDA receptors on RGCs. In contrast to AMPA and kainate receptors (KAR), which predominantly facilitate the movement of sodium (Na⁺) and potassium (K⁺) ions, NMDA receptors permit the influx of calcium ions (Ca²⁺), thereby playing a pivotal role in excitotoxicity. Prolonged or excessive activation of NMDA receptors leads to a sustained influx of calcium ions into the soma of RGCs. This calcium overload triggers an excitotoxic cascade, which includes the degradation of cytoskeletal and membrane proteins, resulting in cellular structural damage. Additionally, it causes DNA fragmentation, which induces apoptosis. The activation of phospholipase A2 further decomposes membrane phospholipids, leading to the production of pro-inflammatory molecules and subsequent membrane damage 85-88.

#### Alterations in CaMKII and calcium homeostasis

The RGCs death following glaucomatous optic nerve damage is associated with dysregulated calcium ion activation. The Ca2+/CaMKII complex serves as a critical regulatory protein within the calcium signaling pathway. In the normal retina, CaMKII is extensively phosphorylated, and its inhibition results in RGC death 89, 90. Activation of the CaMKII/CREB pathway not only provides significant protection to RGC bodies and axons against excitotoxic and optic nerve damage, but also safeguards the long-distance projection of RGC axons from the retina to the visual relay center in the brain. Consequently, this pathway preserves the integrity of the entire visual pathway and maintains visual function 91, 92. A recent study by a research team has developed a nanodrug (HNLO-NPs). This nanodrug is engineered to dissociate and release nicotinamide and oleic acid in response to RGCs experiencing hypoxic conditions and elevated reactive oxygen species (ROS). The released compounds subsequently activate the CaMKII/CREB signaling pathway, thereby conferring protection against RGC apoptosis. This innovative approach may offer a novel perspective for neuroprotective strategies in the treatment of glaucoma 93.

#### Mitochondrial metabolism of RGCs

RGCs possess long axons and are integral to the transmission of visual information; consequently, they necessitate adequate levels of adenosine triphosphate (ATP) to fulfill their elevated energy demands. Mitochondria are crucial for sustaining RGC metabolism by facilitating ATP production through regulating mitochondrial dynamics and oxidative phosphorylation (OXPHOS). When the OXPHOS system is compromised, it leads to the generation of excessive ROS, which in turn induces oxidative stress 94. The disruption of mitochondrial cristae architecture, cytoskeletal degradation, and the accumulation of autophagic profiles observed in the RGCs of DBA/2J mice indicate a deficiency in mitophagy activity. This impairment may further exacerbate mitochondrial dysfunction, contributing to the degeneration of RGCs in the context of glaucoma pathology 95, 96. Elevated IOP contributes to an imbalance in mitochondrial fission and fusion processes, with substantial increases in mitochondrial fission rates observed in experimental models of glaucoma-induced ocular hypertension. This imbalance results in the fragmentation and dysfunction of mitochondria within the somas and axons of RGCs. Research has demonstrated that Apolipoprotein A-I binding protein (AIBP) plays a crucial role in maintaining the integrity of mitochondrial structure and function in RGCs. Elevated intraocular pressure has been shown to significantly reduce AIBP levels in RGCs of DBA/2J mice. This deficiency in AIBP not only impairs the OXPHOS system but also adversely affects mitochondrial dynamics in RGCs, resulting in pronounced mitochondrial fragmentation and the formation of autophagosomes 97, 98.

#### Steady state of potassium ion (K+)

Dysregulation of potassium (K+) homeostasis and channel expression plays a significant role in modulating neuronal excitability in neurodegenerative diseases, such as glaucoma. In experimental models of glaucoma, it has been observed that RGCs undergo an adaptive process in response to sustained elevated intraocular pressure, characterized by diminished potassium ion sensitivity and decreased excitability. RGCs subjected to elevated intraocular pressure exhibit reduced susceptibility to high potassium concentrations, sustaining prolonged spiking activity at increased magnitudes of depolarizing current prior to attaining the threshold for depolarization block 99. Of coruse, the adaptation of RGCs to prolonged stress involves not only intrinsic cellular mechanisms but also significant alterations in the expression of voltage-gated ion channels and the regulation of glial cells in the extracellular milieu. The regulation of extracellular potassium (K+) in the retina is primarily mediated by Müller glial cells. Elevated intraocular pressure can compromise the integrity of the Müller cell plasma membrane, resulting in disruptions of cation homeostasis and subsequent electrophysiological impairment of RGCs 100, 101.

### Transsynaptic degeneration of RGCs associated with glaucoma

The annual rate of RGC loss due to aging is approximately 0.4%; however, this rate increases to 4% in individuals diagnosed with glaucoma 102. Synaptic transmission within the retina primarily takes place at two distinct levels: the bipolar cells and RGCs of the inner retina, and the photoreceptors of the outer retina. At both levels, excitatory signaling is modulated by interneurons that utilize GABAergic and glycinergic neurotransmission 81. The maintenance of ion gradients across the neuronal membrane, essential for optimal neurotransmission, necessitates high energy consumption and intense metabolic activity within axons. These bioenergetic demands are particularly heightened in sustaining the lengthy axons of interneurons, ultimately leading to increased susceptibility to axonal fragility.

Transsynaptic degeneration, a phenomenon wherein primary neuronal damage affects distal neurons via synaptic connections, is referred to as Wallerian degeneration 103. This process is considered the primary mechanism underlying distal axonal degeneration in glaucoma and was first identified and named by Augustus Waller. It is characterized by the disconnection of axons from the neuronal cell body, accompanied by the swelling and degeneration of organelles. Speculation exists that axonal segments within locally swollen areas may signify regions of impeded or disrupted axonal transport, which could potentially lead to a reduced delivery of neurotrophic factors to RGCs. Nonetheless, empirical evidence is currently lacking to support the notion that the direct loss of neurotrophic factors precipitates axonal degeneration 104, 105. In a recent investigation utilizing a mouse model of primary open-angle glaucoma (POAG), researchers identified that axonal degeneration precedes the structural and functional decline of RGCs within the pathological sequence. Notably, the majority of anterograde axonal transport is maintained during the initial phases of axonal degeneration. Progressive impairments in axonal transport and complete defects in optic nerve head transport are observed only during the advanced stages of severe axonal degeneration 106. It is widely accepted that there exists a strong correlation between elevated intra-axonal calcium levels and mitochondrial dysfunction in the initiation of axonal damage during the early stages of glaucoma. The influx of calcium prompts its uptake and subsequent accumulation within the mitochondrial matrix, leading to mitochondrial swelling, membrane rupture, and ultimately resulting in energy depletion 107-109. The progressive accumulation of mitochondrial dysfunction and the concomitant decline in function with advancing age may significantly contribute to the increased susceptibility to age-related glaucoma.

### Axonal transport impairments

Axonal transport is crucial for maintaining nutrient homeostasis and facilitating neurotransmission within the organism. This process encompasses both anterograde transport, which moves materials from the cell body to the axon terminal, and retrograde transport, which conveys materials from the axon terminal back to the cell body. Disruptions in the energy supply for transport and alterations in the structure of dynein and tubulin proteins involved in transport can result in axonal transport dysfunction, impacting the movement of organelles, synaptic components, trophic factors, and other essential molecules, ultimately leading to neurodegenerative diseases. Axonal transport abnormalities are typically identified in the early stages of many neurodegenerative diseases and frequently occur prior to the onset of structural deterioration 110. The blockade of anterograde and retrograde axonal transport in the optic nerve has been extensively documented in experimental glaucoma animal models. This disruption is hypothesized to contribute significantly to the degeneration of RGCs in glaucoma, primarily due to the insufficient delivery of neurotrophic factors and other critical molecules 111-113. Alterations in the cytoskeleton or associated proteins may act as early biomarkers of a degenerative condition, even though they may not immediately impair axonal function 114.

Due to the extensive length of their axons, RGCs primarily rely on long-distance axonal transport mechanisms. The retina, particularly the optic nerve head where RGCs are unmyelinated, exhibits substantial energy demands. Consequently, RGCs are exceptionally sensitive to the energy supply necessary for the propagation of action potentials. In the context of glaucomatous neurodegeneration, extended exposure to adverse conditions such as energy deficiency and persistent neurotoxicity can precipitate a series of anterograde and retrograde transport dysfunctions. Nicotinamide mononucleotide adenylyltransferase 2 (NMNAT2) plays a pivotal role in maintaining axonal integrity, as it is synthesized in the soma and requires continuous axonal transport to ensure an adequate supply of axonal NAD 115. Recent research has demonstrated a significant reduction in NMNAT2 levels in mice afflicted with glaucoma or ocular hypertension. The diminished levels of NAD can be ameliorated through the upregulation of Nicotinamide mononucleotide adenylyltransferase 1 (NMNAT1) or NMNAT2, thereby preserving visual function in glaucomatous mice 116. These findings imply that reduced NMNAT2 levels may play a role in neurodegeneration, while augmenting NMNAT2 and NAD levels could offer a promising therapeutic approach for the treatment of glaucoma and other axonal pathologies 117. Recent research has elucidated the pivotal role of Kif5A in the regulation of neurodegenerative disorders, including glaucoma, as demonstrated through quantitative proteomics. The knockdown of Kif5A has been shown to hinder mitochondrial movement and transport, thereby impairing the axonal transport mechanisms of RGCs 118, 119. Given the dynamic nature of axonal transport, radiographic tracking technology involving intravitreal injection of radioactively labeled substances has been widely employed. Currently, the intraocular injection of cholera toxin B subunit (CTB) conjugated with a fluorescent marker is used to investigate axonal transport. This model is deemed ideal for studying optic neuropathy, as it effectively demonstrates active anterograde transport to the lateral geniculate nucleus and the superior colliculus 120.

## Protecting and regenerating the nerves in glaucoma-related brain damage

### Currently recognized neuroprotective pharmaceuticals

#### Memantine

As a neurodegenerative disease, glaucoma is currently undergoing more and more extensive research on treatment methods from the perspective of preventing or slowing down the damage and loss of RGCs. Memantine, which has received endorsement from various international organizations, is frequently prescribed for patients with moderate to severe Alzheimer's disease and Parkinson's disease. Previous research has demonstrated that elevated concentrations of excitatory glutamate exert neurotoxic effects on RGCs, resulting in increased intracellular calcium levels and subsequent cell death. Memantine, a non-competitive antagonist of NMDA receptors, has been shown to effectively inhibit excessive glutamatergic activity while preserving normal synaptic transmission 121. Preliminary findings indicate that memantine may serve as an effective therapeutic agent for glaucoma. Oral administration of memantine in monkeys with experimentally induced glaucoma has demonstrated a reduction in retinal ganglion cell (RGC) loss compared to untreated control subjects 122. Additionally, ongoing research and development efforts are focused on topical formulations of memantine encapsulated in PLGA-PEG nanoparticles (MEM-NP). These formulations are being evaluated in experimental glaucoma models, with the aim of mitigating RGC damage while enhancing the safety and tolerability of the treatment 123. However, human clinical trials evaluating the neuroprotective efficacy of memantine have produced inconclusive outcomes, necessitating further investigation and discourse 124.

#### Brimonidine

Brimonidine, a selective adrenergic α2 receptor agonist widely utilized in the clinical management of glaucoma, has been acknowledged for its efficacy in reducing intraocular pressure. Recent research has indicated that brimonidine significantly improves the survival rate of RGCs across various animal models. The neuroprotective mechanisms of brimonidine are posited to involve the activation of α2-adrenergic receptors, the upregulation of brain-derived neurotrophic factor, and the engagement of Trk-MAPK signaling pathways. Furthermore, brimonidine has been demonstrated to modulate the electrophysiological responses of RGCs via the MAPK/ERK and PI3K signaling pathways, elevate levels of basic fibroblast growth factor, and inhibit the release of excitatory glutamate, among other effects. Some studies have even found that brimonidine can confer neuroprotection against the loss of RGCs following optic nerve compression 125-128.

### Potential neuroprotective agent

#### Exogenous neurotrophic factors

The neurotrophin family consists of a group of pleiotropic small secreted peptides that play crucial roles in neuronal growth, development, and survival. In mammals, this family encompasses nerve growth factor (NGF), brain-derived neurotrophic factor (BDNF), neurotrophin-3 (NT-3), and neurotrophin-4/5 (NT-4/5) 129. Neurotrophins are recognized for their critical involvement in numerous cellular processes, including differentiation, proliferation, axonal growth, and synaptic transmission. Furthermore, they play a pivotal role in cell development and survival. The role of neurotrophic factors (NTFs) in sustaining cell survival within the retina has been substantiated across various neurodegenerative diseases. Among these factors, Brain-Derived Neurotrophic Factor (BDNF) has garnered significant attention due to its distinctive protective effects on RGCs. BDNF is expressed within the central nervous system and has profound implications for synaptic and structural plasticity, which are essential for maintaining neural integrity 130, 131. Neurotrophic factors within the body engage with two distinct receptor types: tropomyosin receptor kinases (Trks) and p75 neurotrophin receptors (p75NTR). These receptors are distributed across the retina, optic nerve, and visual cortex of the brain. Trk receptors are implicated in promoting axonal growth and inhibiting apoptosis, whereas p75NTR receptors initiate pro-apoptotic signaling pathways, culminating in cellular death 129, 132, 133.

Brain-derived neurotrophic factor (BDNF) exhibits neuroprotective effects through its interaction with tropomyosin receptor kinase B (TrkB) expressed in RGCs and glial cells. BDNF is synthesized in cells located within the retinal neuropil layer and inner nuclear layer. Additionally, it can be transported to the retina from higher visual centers, such as the superior colliculus (SC) and lateral geniculate nucleus (LGN), via retrograde axonal transport. Furthermore, BDNF can be conveyed within the SC and LGN through RGC axons, enabling bidirectional transport between these regions. The initial identification of the neuroprotective effects of Brain-Derived Neurotrophic Factor (BDNF) was characterized by its direct modulation of RGCs, which promotes neuronal survival within the retina 134. Subsequent research has consistently demonstrated that the disruption of BDNF signaling in glaucoma contributes to apoptosis and degeneration of RGCs, primarily due to inadequate nutritional support 135. Brain-derived neurotrophic factor (BDNF) is postulated to regulate the synthesis of critical proteins implicated in synaptic plasticity. This complex regulatory mechanism has been demonstrated to promote neuronal survival post-injury across various neurodegenerative conditions 136. For example, empirical evidence suggests that the reduction of BDNF levels in Alzheimer's disease is significantly correlated with amyloid accumulation, tau hyperphosphorylation, and neuronal apoptosis. Consequently, modulating the BDNF signaling pathway holds promise for ameliorating cognitive deficits in individuals afflicted with Alzheimer's disease 137-139. And in patients with Parkinson's disease, a significant reduction in the expression of BDNF/TrkB within the substantia nigra has been observed. Conversely, activation of the BDNF pathway has been shown to markedly enhance nerve regeneration and exhibit substantial anti-apoptotic effects 140, 141.

**Figure 3:**
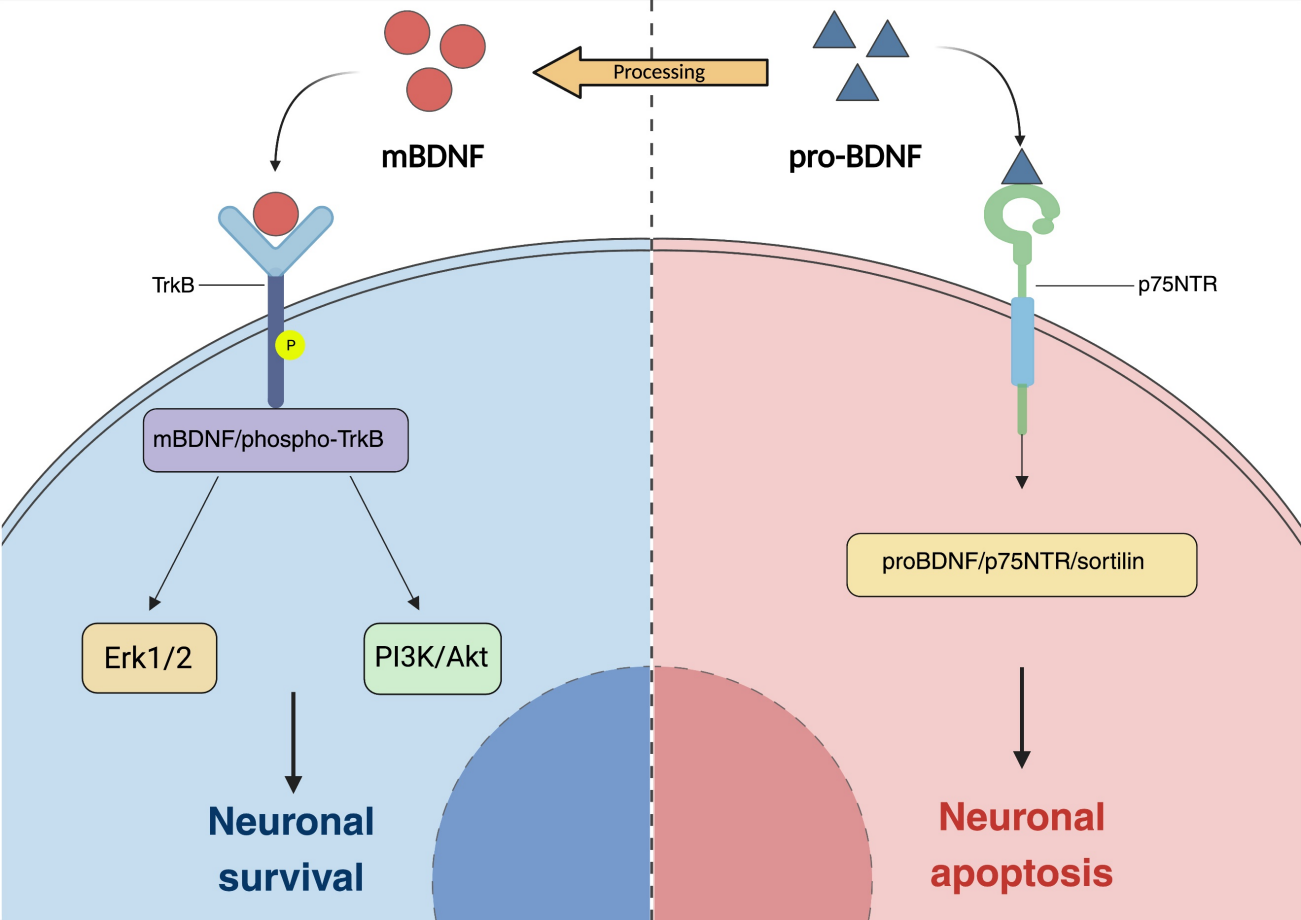
** The underlying mechanism through which BDNF confers neuroprotective effects.** (a) Production and secretion of brain-derived neurotrophic factor (BDNF). BDNF is synthesized and secreted by cells in the neuropil layer and the inner nuclear layer of the retina. A significant portion of BDNF is transported retrogradely from neural cells in the brain to the retina. BDNF exerts its effects by directly binding to the tropomyosin receptor kinase B (TrkB) expressed in retinal ganglion cells (RGCs) and/or indirectly binding to TrkB expressed in Müller glial cells, thereby facilitating the survival of neuronal cells. In glaucoma, elevated intraocular pressure impedes the retrograde transport of BDNF from the brain, resulting in damage to RGCs. (b) BDNF can be activated in two distinct forms: the prodomain of BDNF (pro-neurotrophin isoform of BDNF, pro-BDNF) and the mature isoform of BDNF (mBDNF), these forms primarily exert their functions through distinct signaling pathways. ProBDNF interacts with p75NTR and the transport protein sortilin to form a proBDNF/p75NTR/sortilin binding complex, which subsequently induces neuronal cell apoptosis. In contrast, proBDNF is processed into its mature form, mBDNF, which exhibits a high affinity for TrkB. This interaction triggers autophosphorylation and dimerization of TrkB, thereby activating downstream signaling pathways that are crucial for neuronal survival and plasticity. BDNF brain-derived neurotrophic factor, TrkB tropomyosin receptor kinase B, RGCs retinal ganglion cells, p75NTR p75 neurotrophin receptor.

Upon activation of TrkB receptors, BDNF initiates various signaling pathways in the retina, including the extracellular signal-regulated kinase 1/2 (Erk1/2) and phosphatidylinositol 3-kinase (PI3K)/Akt pathways. In adult RGCs, activation of TrkB receptors leads to stimulation of both pathways. Oddone identified that individuals diagnosed with open-angle glaucoma demonstrate significantly lower serum concentrations of brain-derived neurotrophic factor (BDNF) in comparison to healthy controls. Furthermore, these neurotrophic factor levels exhibit a substantial correlation with the mean deviation observed in visual field assessments 142. In an initial investigation, the direct injection of brain-derived neurotrophic factor (BDNF) into the superior colliculus of neonatal hamsters resulted in a 13- to 15-fold reduction in retinal ganglion cell (RGC) loss. Subsequent research has consistently demonstrated that the exogenous administration of BDNF or TrkB agonists enhances RGC survival across both acute and chronic models of optic nerve injury 143-146. The administration of human BDNF eye drops in an animal model of chronic ocular hypertension demonstrates potential in restoring deficits in patterned electroretinogram (P-ERG) and visual cortex evoked potential (VEP), thereby further substantiating the neuroprotective properties of BDNF 147 (Fig. 3).

Recent research on novel adeno-associated virus (AAV) gene therapy targeting RGCs has demonstrated dual functionality: it enhances brain-derived neurotrophic factor (BDNF) production by targeting astrocytes and Müller cells, and it activates the BDNF receptor (TrkB) expression in the retina, thereby promoting long-term neuroprotective signaling 135. The potential for gene-targeted supplementation of BDNF and other neurotrophic factors to mitigate metabolic burden and compensate for the inadequate transport of trophic factors resulting from axonal damage in human glaucoma warrants further investigation 145, 148.

#### Erythropoietin (EPO)

Erythropoietin, a glycoprotein cytokine predominantly produced in the kidneys, was originally recognized for its capacity to stimulate erythropoiesis by promoting the proliferation and differentiation of hematopoietic progenitor cells in the bone marrow. Recent studies, however, have elucidated its neuroprotective effects within the central nervous system 149. The central nervous system (CNS) produces minimal amounts of erythropoietin (EPO), with its receptors expressed on various CNS cell types, including neurons and glial cells. Several prior reviews have examined the anti-apoptotic role of erythropoietin (EPO) in central nervous system diseases. These studies suggest that EPO may mitigate neuroinflammation by reducing reactive oxygen species, inhibit microglial cell proliferation by decreasing cellular reactivity, and thereby indirectly attenuate chronic neuroinflammation 150-152. Studies have demonstrated that systemic administration of erythropoietin (EPO) in animal models of induced Parkinson's disease yields a pronounced neuroprotective effect. Furthermore, Parkinson's disease patients treated with recombinant human EPO (rhEPO) exhibited significant amelioration of non-motor symptoms in comparison to those receiving a placebo 153, 154. In a murine model of Alzheimer's disease (AD), intraperitoneal administration of recombinant human erythropoietin (EPO) resulted in significant improvements in memory function relative to saline-treated controls. Additionally, EPO treatment was observed to restore serotonin receptor levels. These findings suggest that EPO may exert anti-inflammatory effects through activation of the serotonergic pathway, positioning it as a potential therapeutic agent for AD 155.

Analogous to its role in the central nervous system (CNS), erythropoietin (EPO) has demonstrated neuroprotective effects in RGCs 137, 138. A study investigating the survival rate of primary RGCs revealed that EPO confers neuroprotection comparable to brain-derived neurotrophic factor (BDNF) in reducing RGC death induced by glutamate and nitric oxide (NO) toxicity 156. Concurrently, the ability of erythropoietin (EPO) to penetrate the blood-brain barrier, as evidenced by the increased survival of RGCs following intravitreal administration of EPO in an optic nerve transection model, provides compelling evidence for the neuroprotective and neuroregenerative properties of EPO 157. In recent years, there has been an increasing interest in gene-based protective therapies. Numerous studies have employed adeno-associated virus (AAV) vectors to deliver erythropoietin (EPO), while recombinant adeno-associated virus (rAAV) constructs have been utilized to introduce genes that attenuate erythropoiesis. The administration of the active form of erythropoietin (EpoR76E) has demonstrated promising outcomes in decreasing the expression of pro-inflammatory cytokines and inhibiting microglial reactivity in DBA/2J mice 158. And the researcher posits that EpoR76E is safer than the wild-type erythropoietin (EPO) and does not induce an increase in hematocrit levels. Furthermore, a notable advantage of EpoR76E treatment is its ability to preserve microglial reactivity. In other experimental models, the complete inhibition of microglial responses has been shown to exacerbate neurodegeneration. Therefore, modulating microglial reactivity is crucial for neuroprotection 159, 160.

### Restoration of axonal transport

**Figure 4:**
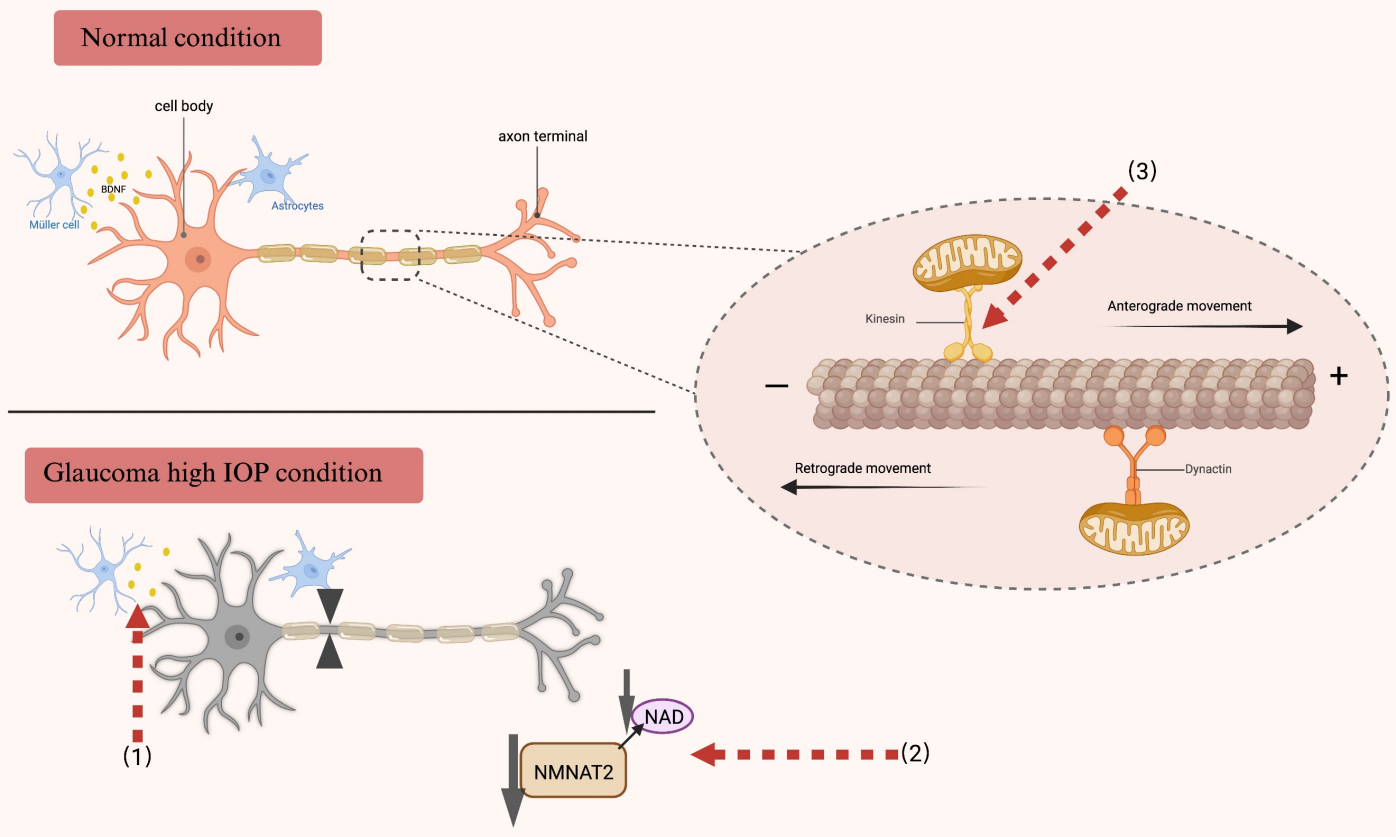
** Axonal transport under normal physiological conditions and in individuals diagnosed with glaucoma.** RGCs necessitate an adequate energy supply to preserve the integrity of axonal transport. The motor proteins kinesin and dynein, in conjunction with microtubules, facilitate the transport of essential substances and organelles necessary for this process. In glaucoma, the disruption of neuroprotective factor transport, inadequate energy supply, and impaired mitochondrial transport may contribute to neurodegeneration. Red arrows denote therapeutic targets for addressing impaired axonal transport. 1 Disruption of BDNF transport and a decrease in its production have been documented in glaucoma, and supplementation with BDNF may promote axonal transport and postsynaptic neuron regeneration after retinal injury. 2 NAD serves as a critical substrate for energy metabolism, primarily synthesized by NMNAT2. The maintenance of NAD homeostasis is essential for retinal health. Glaucoma induces a reduction in NMNAT2 levels within the long axons of RGCs, leading to impaired axonal transport. Consequently, therapeutic strategies aimed at axonal protection in glaucoma should focus on restoring NAD levels and modulating NMNAT2 metabolism. 3 The maintenance of RGC survival and functionality is critically dependent on mitochondrial transport. In glaucoma, this transport is disrupted, adversely affecting axonal transport and resulting in progressive degeneration. The expression of the kinematic protein Kif5A, which regulates the anterograde transport of mitochondria, may represent a novel approach to enhance the functional regeneration of RGCs following injury. BDNF brain-derived neurotrophic factor, RGCs retinal ganglion cells, NAD nicotinamide adenine dinucleotide, NMNAT2 Nicotinamide mononucleotide adenylyltransferase 2.

In glaucoma models, axonal transport is modulated by a range of factors, including elevated intraocular pressure, age, metabolic processes, and alterations in gene expression. The restoration of axonal transport was initially demonstrated to be advantageous in the disease process of primates experiencing short-term elevated intraocular pressure. Furthermore, the obstruction of axonal transport was promptly reversed upon normalization of intraocular pressure 161. As previously elucidated, the neuroprotective effects of brain-derived neurotrophic factor (BDNF) in a rat model of glaucoma are mediated through the enhancement of both anterograde and retrograde axonal transport via intravitreal administration of BDNF. This intervention results in elevated levels of motor proteins and kinesin within the optic nerve. Beyond the role of neurotrophic factors, modulation of axonal transport may also be accomplished by augmenting energy supply. Diminished ATP production and compromised mitochondrial function have been implicated in the pathogenesis of various neurodegenerative disorders 162. NAD+/NADH is integral to numerous metabolic pathways and cellular signaling processes that are mediated by ATP. Enhancing NAD+ concentrations has demonstrated neuroprotective effects in various neurodegenerative diseases, such as Alzheimer's disease and Parkinson's disease 163-166. In experimental models of glaucoma, elevated concentrations of metabolic substrates demonstrate a protective effect against axonal degeneration. Furthermore, the modulation of nicotinamide adenine dinucleotide (NAD) metabolism has been shown to enhance axonal energy reserves and facilitate the maintenance of axoplasmic flow in RGCs. The Wallerian degeneration slow (WldS) allele encodes a chimeric protein with NAD-related activity, which has been shown to enhance the function and viability of compromised mitochondria. The WldS protein, along with other related WLD proteins, presents potential therapeutic advantages in providing protection against a range of neurodegenerative disorders, including glaucoma 167-169. In DBA/2J mice, the concurrent administration of nicotinamide (a precursor to NAD) and WLD markedly mitigated glaucomatous axonal degeneration, synaptic loss, and mitochondrial alterations. Moreover, the study revealed that the synergistic effects of these compounds provided enhanced protection against axonal transport impairment in comparison to the administration of individual treatments 170 (Fig. 4). Furthermore, Stankowska and her research team elucidated the protective properties of a short peptide on RGCs. Specifically, they discovered that αB-crystallin (HSPB5), a member of the small heat shock protein family, demonstrated chaperone and anti-apoptotic characteristics analogous to its precursor within the core domain short peptide D73RFSVNLDVKHFSPEELKVK92 (peptain) 171. In a murine model of ocular hypertension, the intravitreal administration of peptain-1 and peptain-3a was observed to attenuate retinal ganglion cell (RGC) degeneration and ameliorate impairments in axonal transport. Nevertheless, the precise mechanism by which this intervention preserves axonal transport remains uncertain, with speculation suggesting a potential protective role on the cytoskeleton. Additional investigations are warranted to elucidate this mechanism further 172, 173. These results provide evidence that improving axonal transport can be an effective neuroprotective approach, offering advantages in preserving the survival and functionality of RGCs, as well as in sustaining the connections between the retina and the brain.

### Regeneration of axons in the optic nerve

Improving the viability of RGCs is a crucial aspect of glaucoma neuroprotection strategies. In addition to cell apoptosis prevention, an ideal neuroprotective approach for glaucoma should also focus on stimulating RGC axonal regeneration to facilitate the reconstruction of connections with the brain. The mTOR pathway, which is active during development but downregulated in the mature nervous system, is further suppressed following injury. Consequently, numerous academic studies have concentrated on investigating the role of the mTOR pathway in facilitating the regeneration of retinal ganglion cell axons. Research has demonstrated that the upregulation of mTOR signaling plays a crucial role in promoting the survival of RGCs and facilitating axon regeneration following optic nerve injury. Phosphatase and Tensin Homologue gene (PTEN) functions as a key negative regulator of the mTOR pathway. Numerous studies have corroborated that mice lacking phosphatase and tensin homologue PTEN exhibit nerve fiber regeneration and partial restoration of visual functions 174, 175. Moreover, SOCS3, a cytokine signaling molecule known for its role as a negative regulator of the JAK/STAT3 pathway, has been shown to promote substantial axon regeneration and amplify the regenerative benefits of Ciliary Neurotrophic Factor (CNTF). It is important to acknowledge that while individual deletions of PTEN or SOCS3 demonstrated substantial regenerative capabilities, their effects diminished over time following optic nerve injury. Consequently, this study sought to assess the collective impact of deleting both genes on axonal regeneration in mice with optic nerve damage. The findings revealed that the synergistic effect of co-deletion led to a more than tenfold increase in the quantity of regenerated axons compared to the deletion of either gene alone, contributing to the establishment of synapses in the suprachiasmatic nucleus 175-177. Similarly, a more enhanced axonal regeneration outcome could potentially be attained through the utilization of a combination of exogenous neurotrophic factors to optimize signal transduction and upregulation facilitated by multiple pathways.

**Figure 5:**
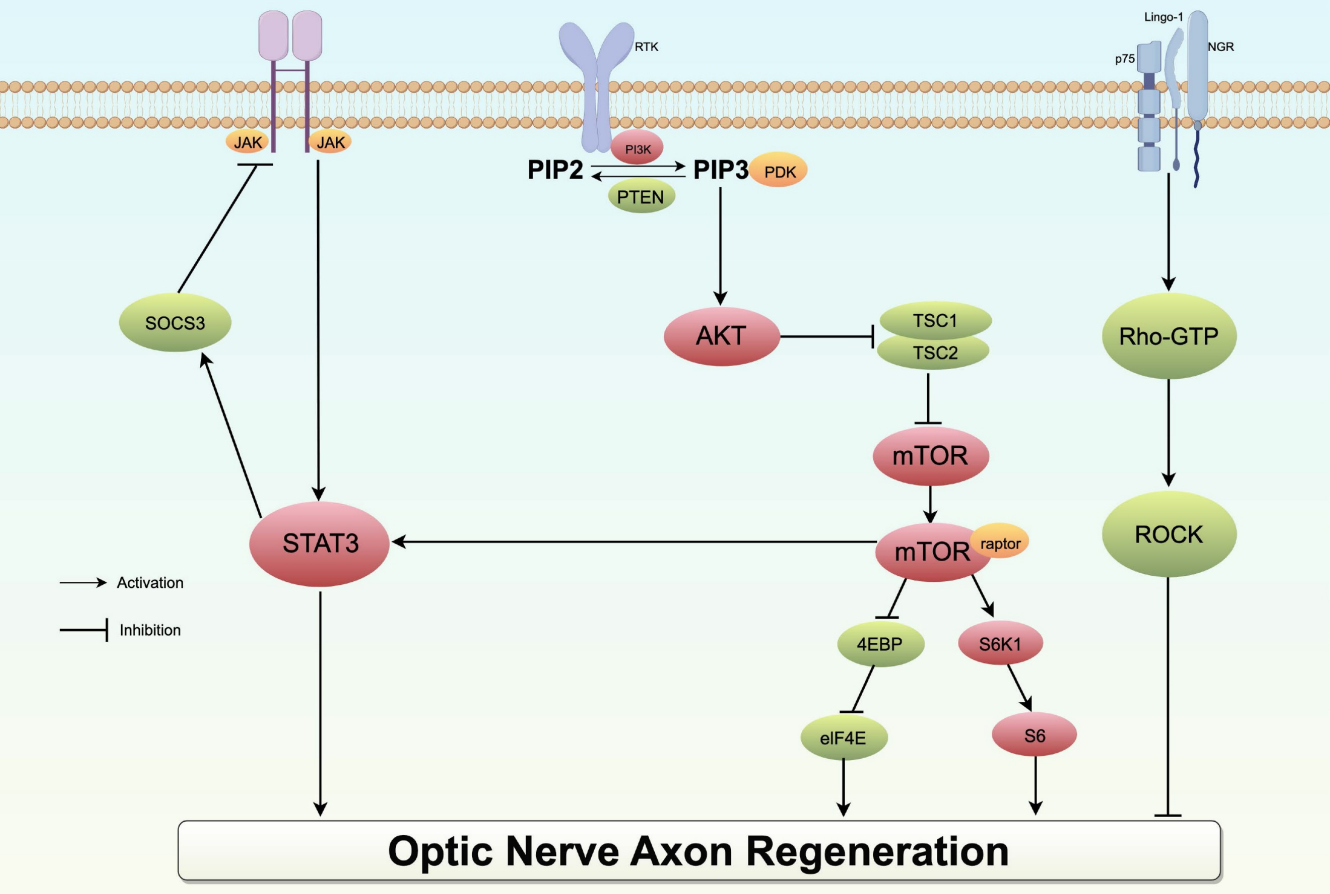
** Schematic diagram of the PI3K/AKT/mTOR, JAK/STAT3 and Rho pathway in optic nerve axon regeneration.** In the STAT/JAK signaling pathway, the application of the neurotrophic factor CNTF or the removal of SOCS3, a STAT inhibitor, can activate the pathway, thereby promoting axon regeneration. Within the mTOR signaling pathway, PTEN, TSC1, and AKT act as negative regulators; the knockout of these genes can mitigate inhibitory effects and promote axonal regeneration. In contrast, the activation of myelin-associated inhibitors and their corresponding receptors initiates the Rho/ROCK pathway, resulting in the phosphorylation of multiple substrates and ultimately impeding axonal growth.

The mature optic nerve environment hinders axonal regeneration, necessitating the identification of targets for the inhibitory effect in order to promote regeneration. Various inhibitory molecules, such as Nogo-A and myelin-associated glycoprotein (MAG), have been identified, which transmit signals through Rho and ROCK proteins in retinal ganglion cell (RGC) growth cones. Rho, a constituent of the cytoplasmic Rho family of small GTP-binding molecules, is associated with Rho-associated coiled-coil-containing protein kinase (ROCK), which is the primary downstream factor extensively examined in research. Various downstream factors within the Rho-ROCK pathway have been identified as being prominently expressed in the central nervous system. Their phosphorylation has been shown to impede neuronal survival and axon regeneration via the PI3K/AkT/mTOR pathway. Hence, within the natural, fully developed central nervous system milieu, the regeneration of impaired axons is impeded by the binding of certain myelin-related inhibitory signals to high-affinity receptors, thereby activating the Rho-ROCK pathway and inducing growth inhibition. Consequently, ROCK inhibitors have been employed as neuroprotective interventions in a range of neurodegenerative conditions such as glaucoma (Fig. 5). Notably, research has demonstrated that inhibition of the ROCK pathway can mitigate degeneration in Alzheimer's disease, Parkinson's disease, Huntington's disease, among others. The potential of ROCK inhibitors to enhance axonal growth has been demonstrated in both *in vitro* and *in vivo* studies related to glaucoma 178. Tokushige conducted an experiment in which the ROCK inhibitor Y-27632 was administered to primary RGCs, resulting in a notable enhancement of neurite growth and the regeneration of synapses. Furthermore, when Y-27632 was combined with CNTF, the growth trend became even more pronounced 179. Furthermore, an observed trend of increased synapse length was noted in rats with knocking down ROCKII activity, providing additional evidence that inhibiting ROCK can mitigate the inhibitory effects on neurite growth 180. In the rat model of optic nerve crush, administration of Y-27632 alone or in combination with CNTF resulted in a robust regenerative response, with a greater increase in the number of regenerated axons compared to their length 181, 182. Concurrently, certain research studies have indicated that ROCK inhibitors have the potential to enhance blood flow in ONH and may also exhibit a protective effect in the development of glaucoma by influencing blood vessels and perfusion 179.

## Conclusion and future challenges

This review examines alterations in the complete visual pathway, spanning from the retina to the cerebral cortex, in individuals with glaucoma. The review also synthesizes evidence supporting the occurrence of glaucomatous transsynaptic degeneration within the central visual system. While lowering intraocular pressure remains the primary treatment approach for glaucoma, the unique significance of RGCs in glaucoma and their potential relevance to neurodegenerative conditions such as Alzheimer's disease and Parkinson's disease necessitate further consideration. In summary, understanding the mechanisms underlying neuroprotection and nerve regeneration in glaucoma, as elucidated by retinal ganglion cells (RGCs), may provide promising strategies for mitigating disease progression and preserving visual function. Given the strong correlation between glaucoma and neurodegenerative disorders of the central nervous system, extensive research has been conducted on enhancing retinal ganglion cell axonal transport, facilitating regeneration, and elucidating the mechanisms of action of diverse neurotrophic factors. However, it is imperative that neurotrophic medications demonstrating significant efficacy in preclinical investigations undergo rigorous validation through large-scale and efficacious clinical trials prior to their implementation in clinical settings.

Furthermore, the majority of existing research on the regrowth potential of RGCs following injury is primarily focused on the retina or optic nerve. Given its classification as an ocular neurological disorder characterized by extensive transsynaptic degeneration, glaucoma frequently leads to impairment of the neural pathways connecting the eye to the brain, highlighting the interconnected nature of these conditions. Limited knowledge exists regarding the potential impact of postsynaptic targets in the brain on the regeneration and reconnection of retinal ganglion cell axons. Recent research has demonstrated that activating neurons beyond the site of injury in the brain can facilitate axon regrowth. Utilizing targeted approaches, including the stimulation of specific postsynaptic target neurons, may enhance axonal regeneration within distinct neural circuits. Limited knowledge exists regarding the potential impact of postsynaptic targets in the brain on the regeneration and reconnection of retinal ganglion cell axons. In the past, the activation of distally regulated neuronal activity using invasive or non-invasive techniques has demonstrated therapeutic benefits in conditions such as Parkinson's disease, epilepsy, and other disorders. Likewise, in the context of the visual system, such methods have shown promise in ameliorating visual field impairments in individuals with glaucoma. These discoveries hold significant potential for the restoration of vision and the enhancement of visual pathway functionality 183-186.

In recent years, numerous studies have focused on neuroprotective therapies utilizing gene delivery, with gene delivery vectors categorized into viral and non-viral systems. Adeno-associated virus (AAV) vectors are currently the predominant choice for glaucoma gene therapy delivery, encoding genes such as BDNF, EPO, and NRF2, enabling uncontrolled expression of target genes in AAV-transduced cells in both *in vivo* and *in vitro* models. These studies aim to investigate the neuroprotective impact of these therapies on RGCs 135, 145, 187. While viral vector gene delivery holds promise for gene protection therapy in glaucoma, it is important to address safety concerns related to immunogenic responses and heightened inflammation triggered by viral vectors, as well as the substantial costs associated with virus production. Research on non-viral gene delivery systems for the treatment of glaucoma has advanced, yet its implementation is hindered by low efficacy and the presence of intricate barriers within ocular tissues. Certain forms of congenital glaucoma, including Primary Open-Angle Glaucoma (POAG), Juvenile-onset open-angle glaucoma (JOAG), and Axenfeld-Rieger syndrome, have been associated with specific genetic mutations that could potentially inform gene therapy strategies. Several gene-based retinal ganglion cell (RGC) neuroprotective therapies show promise in addressing glaucoma and potentially halting or slowing disease progression. Nevertheless, the safety and efficacy of gene therapy remain significant obstacles. Further investigation and exploration are necessary before progressing to clinical trials.

## Abbreviations

RGCs: retinal ganglion cells
IOP: intraocular pressure
LGN: lateral geniculate nucleus
TSD: trans-synaptic degeneration
MRI: magnetic resonance imaging
SD: OCT spectral domain optical coherence tomography
ONH: optic nerve head
OBF: ocular blood flow
LSFG: Laser Speckle Flowgraphy
NTG: normal-tension glaucoma
OSAS: obstructive sleep apnea syndrome
mtDNA: mitochondrial DNA
POAG: open-angle glaucoma
AD: Alzheimer's disease
mRGCs: melanopsin retinal ganglion cells
AIBP: Apolipoprotein A-I binding protein
NMDA: N-methyl-D-aspartate
AMPA: α-amino-3-hydroxy-5-methyl-4-isoxazolepropionic acid
ROS: reactive oxygen specis
Aβ: amyloid-beta
ONL: outer nuclear layer
NFL: nerve fiber layer
APP: amyloid precursor protein
POAG: open-angle glaucoma
OCT: Optical Coherence Tomography
OCTA: Optical Coherence Tomography Angiography
NMNAT1: Nicotinamide mononucleotide adenylyltransferase 1
NMNAT2: Nicotinamide mononucleotide adenylyltransferase 2
CTB: cholera toxin B subunit
P: parvocellular
M: magnocellular
CBF: cerebral blood flow
ATP: adenosine triphosphate
OXPHOS: oxidative phosphorylation
MEM-NP: PLGA-PEG nanoparticles
NGF: nerve growth factor
BDNF: brain-derived neurotrophic factor
NT-3: neurotrophin-3
NT-4/5: neurotrophin-4/5
NTFs: neurotrophic factors
Trks: tropomyosin receptor kinases
SC: superior colliculus
VEP: visual cortex evoked potential
CNS: central nervous system
EPO: erythropoietin
rhEPO: recombinant human EPO
NO: nitric oxide
rAAV: recombinant adeno-associated virus
AAV: adeno-associated virus
NAD: nicotinamide adenine dinucleotide
WldS: Wallerian degeneration slow
PTEN: Phosphatase and Tensin Homologue gene
CNTF: Ciliary Neurotrophic Factor
BDNF: Brain-derived neurotrophic factor
ROCK: Rho-associated coiled-coil-containing protein kinase
